# Mussel-Inspired Polydopamine-Based Multilayered Coatings for Enhanced Bone Formation

**DOI:** 10.3389/fbioe.2022.952500

**Published:** 2022-07-07

**Authors:** Hao Wu, Cancan Zhao, Kaili Lin, Xudong Wang

**Affiliations:** Department of Oral and Cranio-Maxillofacial Surgery, Shanghai Ninth People’s Hospital, Shanghai Jiao Tong University School of Medicine, College of Stomatology, Shanghai Jiao Tong University, National Center for Stomatology, National Clinical Research Center for Oral Diseases, Shanghai Key Laboratory of Stomatology, Research Unit of Oral and Maxillofacial Regenerative Medicine, Chinese Academy of Medical Sciences, Shanghai, China

**Keywords:** bone regeneration, polydopamine, multilayered coating, multifunction, osteogenesis, immunomodulation

## Abstract

Repairing bone defects remains a challenge in clinical practice and the application of artificial scaffolds can enhance local bone formation, but the function of unmodified scaffolds is limited. Considering different application scenarios, the scaffolds should be multifunctionalized to meet specific demands. Inspired by the superior adhesive property of mussels, polydopamine (PDA) has attracted extensive attention due to its universal capacity to assemble on all biomaterials and promote further adsorption of multiple external components to form PDA-based multilayered coatings with multifunctional property, which can induce synergistic enhancement of new bone formation, such as immunomodulation, angiogenesis, antibiosis and antitumor property. This review will summarize mussel-inspired PDA-based multilayered coatings for enhanced bone formation, including formation mechanism and biofunction of PDA coating, as well as different functional components. The synergistic enhancement of multiple functions for better bone formation will also be discussed. This review will inspire the design and fabrication of PDA-based multilayered coatings for different application scenarios and promote deeper understanding of their effect on bone formation, but more efforts should be made to achieve clinical translation. On this basis, we present a critical conclusion, and forecast the prospects of PDA-based multilayered coatings for bone regeneration.

## Introduction

Mussels are promiscuous fouling organisms, which are able to secret adhesive proteins to attach themself to actually all kinds of surfaces, including materials with classical adhesion-resistant property like poly(tetrafluoroethylene) (PTFE) (Waite and Tanzer, 1981; Lee et al., 2007). Waite and Qin discovered that the proteins near the interface of plaque and substrate were rich in 3,4-dihydroxy-L-phenylalanine (dopamine, DA), and the amino acid composition might result in mussels’ adhesive property (Waite and Qin, 2001). The complex zwitterionic structure of DA contains basic amino groups and acidic catechol, which can oxide in aqueous solution of alkaline environments to form a polymer-like film named polydopamine (PDA) on actually all biomaterial surfaces (Salomaki et al., 2018). Through such reaction, the bulk solidification of DA can be achieved with adhesive property, showing outstanding covalent and noncovalent interactions with modified substrates (Yu et al., 1999; Sever et al., 2004; Lee et al., 2006).

**FIGURE 1:**
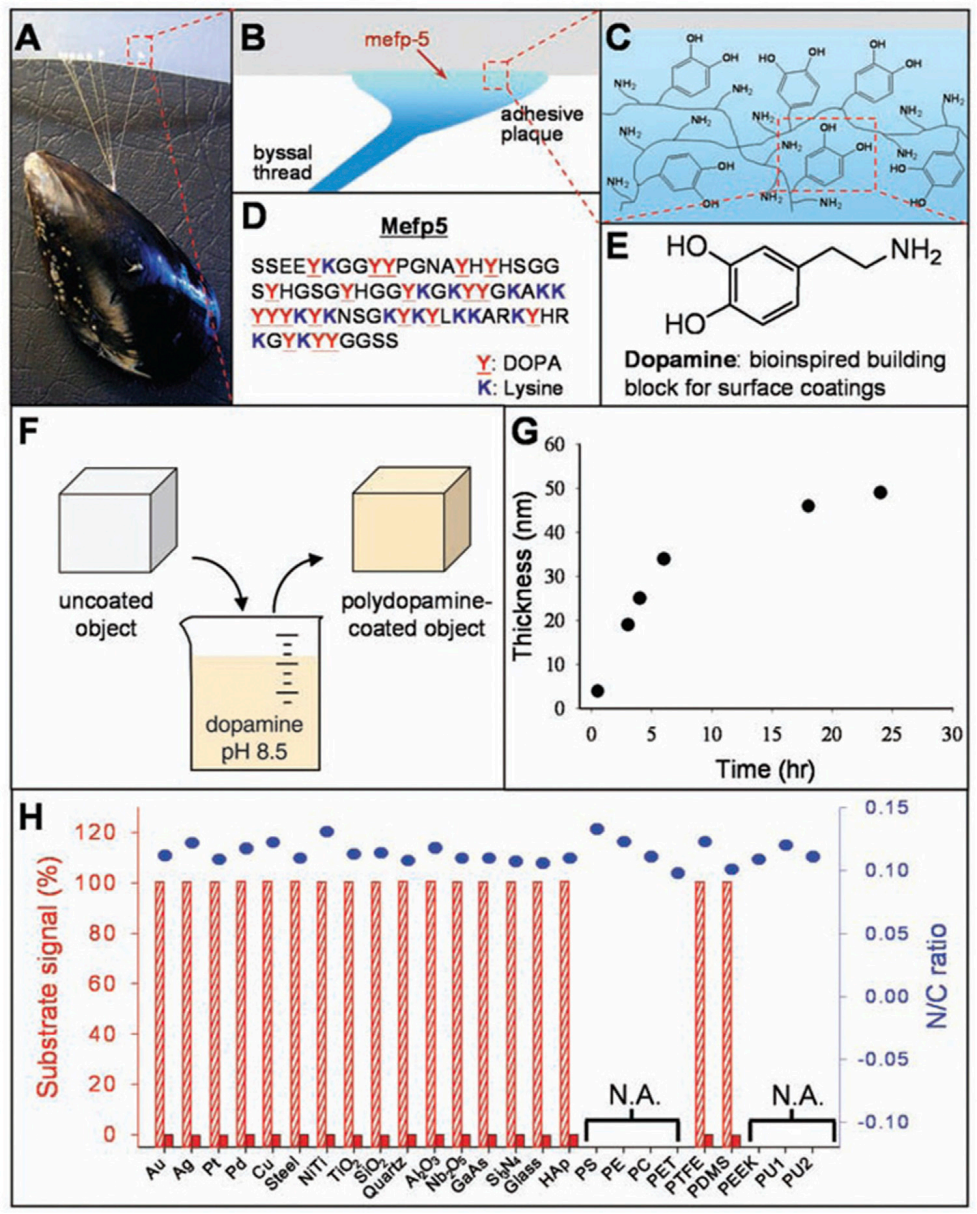
The formation of PDA coatings onto plenty of materials’ surfaces. **(A)** Photograph of a mussel attached to PTFE. **(B**,**C)** Interfacial location of Mefp-5, as well as the representation of catechol and amine groups. **(D)** The sequence of amino acid in Mefp-5. **(E)** DA contains same functional catechol and amine groups. **(F)** A schematic illustration of PDA deposition. **(G)** Thickness of PDA coating. **(H)** XPS evaluation of PDA-coated surfaces (Lee et al., 2007). Copyright 2007, The American Association for the Advancement of Science.

Inspired by mussels’ adhesive property and the composition of adhesive proteins, Lee et al. carried out pioneering work to fabricate PDA films onto plenty of materials’ surfaces, including polymers, ceramics, metals, semiconductors and oxides by self-polymerization (Figure 1) (Lee et al., 2007). It has been demonstrated that through simple immersion in an alkaline aqueous solution of DA, where the pH is buffered to 8.5, similar to marine environments (pH 8.5), an independent thin adherent polymer film can be spontaneously formed on treated substrates by self-polymerization (Wang H. et al., 2019; Wang H. et al., 2021; Augustine et al., 2022). Furthermore, due to the functional groups of PDA coating, bioactive components can be attached to PDA-modified substrates *via* covalent and noncovalent binding, including metallic and polymeric components, ceramics, proteins, peptides, drugs and so on. Therefore, PDA coating can serve as a promising platform for plenty of secondary treatments to create multilayered coatings through the layer-by-layer process, which is potential for functional modification of biomaterials (Youn et al., 2019; Jin et al., 2020; Ma et al., 2020; Zeng et al., 2020; Sun Y. et al., 2021; Tang et al., 2021; Wei et al., 2022).

Caused by injury, tumors, infection, osteonecrosis and other pathological factors, repairing bone defects remains a challenge in clinical practice, which will do harm to patients’ living quality. The application of biomaterial scaffolds has provided an effective proposal for bone regeneration of defected bones (Turnbull et al., 2018; Nikolova and Chavali, 2019). However, the biofunction of unmodified scaffolds is usually limited, which can’t meet demands in specific application scenarios, such as immunomodulation, angiogenesis, antibiosis and so on.

PDA-based multilayered coatings have been proved to endow biomaterials with multifunctional property, showing great potential in many fields including drug delivery, bone tissue repair and regeneration (Bao et al., 2018; Jin et al., 2020; Wu et al., 2020; Zeng et al., 2020; Ling et al., 2021; Su et al., 2021; Ma et al., 2022). After modified by the PDA coating, the substrates will become bioactive, and then the functional groups of PDA can mediate other functional components to form multilayered coatings and make it multifunctional, where the synergistic enhancement of different functions can further promote osteogenic differentiation and new bone formation (Zhang et al., 2018; Wang H. et al., 2019; Wang X. et al., 2019; Kaushik et al., 2020; Sun Y. et al., 2021; Wang H. et al., 2021). Recently, PDA-based multilayered coatings have attracted increasing attention and rapid development has occurred for the related researches of different PDA-based multilayered coatings.

**FIGURE 2:**
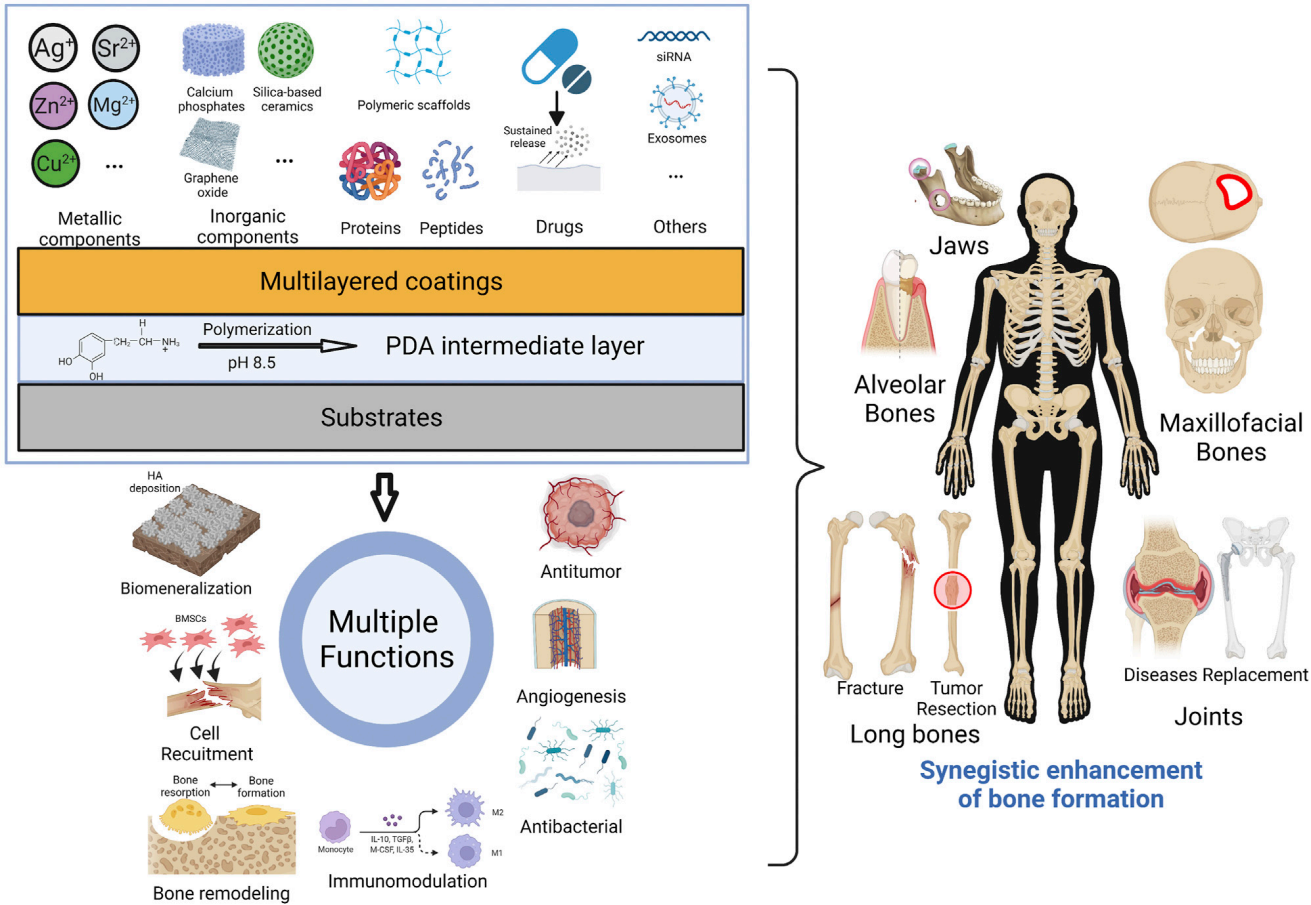
Graphical diagram for fabrication of PDA-based multilayered coatings to achieve multi-functionalization and enhanced bone formation. (Created with BioRender.com).

In this review, we summarize related researches of mussel-inspired PDA-based multilayered coatings, in order to facilitate further design and application for enhanced bone formation. It will be involved in this review about the formation mechanism and biofunction of PDA coating, PDA-mediated different functional components of multilayered coatings, as well as synergistic enhancement of multifunction for new bone formation of bone defects (Figure 2). We also discuss the current challenges and future prospects of PDA-based multilayered coatings on biomaterials for clinical translation. This review might help to develop versatile multifunctional biomaterials in the future to meet the demand for bone repair.

## Formation and Function of PDA Coating

### Formation Approaches for PDA Coating

There are three common approaches for PDA formation, namely enzymatic oxidation, electropolymerization, and solution oxidation, which is most widely used (Liu et al., 2014). Once the DA hydrochloride is dissolved in an alkaline solution and exposed to atmospheric oxygen, the self-polymerization will begin immediately without any strict reaction conditions (Bernsmann et al., 2011). However, after solution oxidation, the uniformity and thickness of the PDA coating may be unsatisfactory, resulting from deposited insoluble precipitate during the solution oxidation. The electropolymerization method is able to achieve a desired coating thickness of PDA, but its requirement of conductivity limits its application (Batul et al., 2017). The enzymatic oxidation for fabrication of PDA coating exhibits the advantage of higher polymerization rate and better stability, but its simplicity is not so good as solution oxidation and still needs more studies for extended applications (Li F. et al., 2018).

### Formation Mechanism of PDA Coating

**FIGURE 3:**
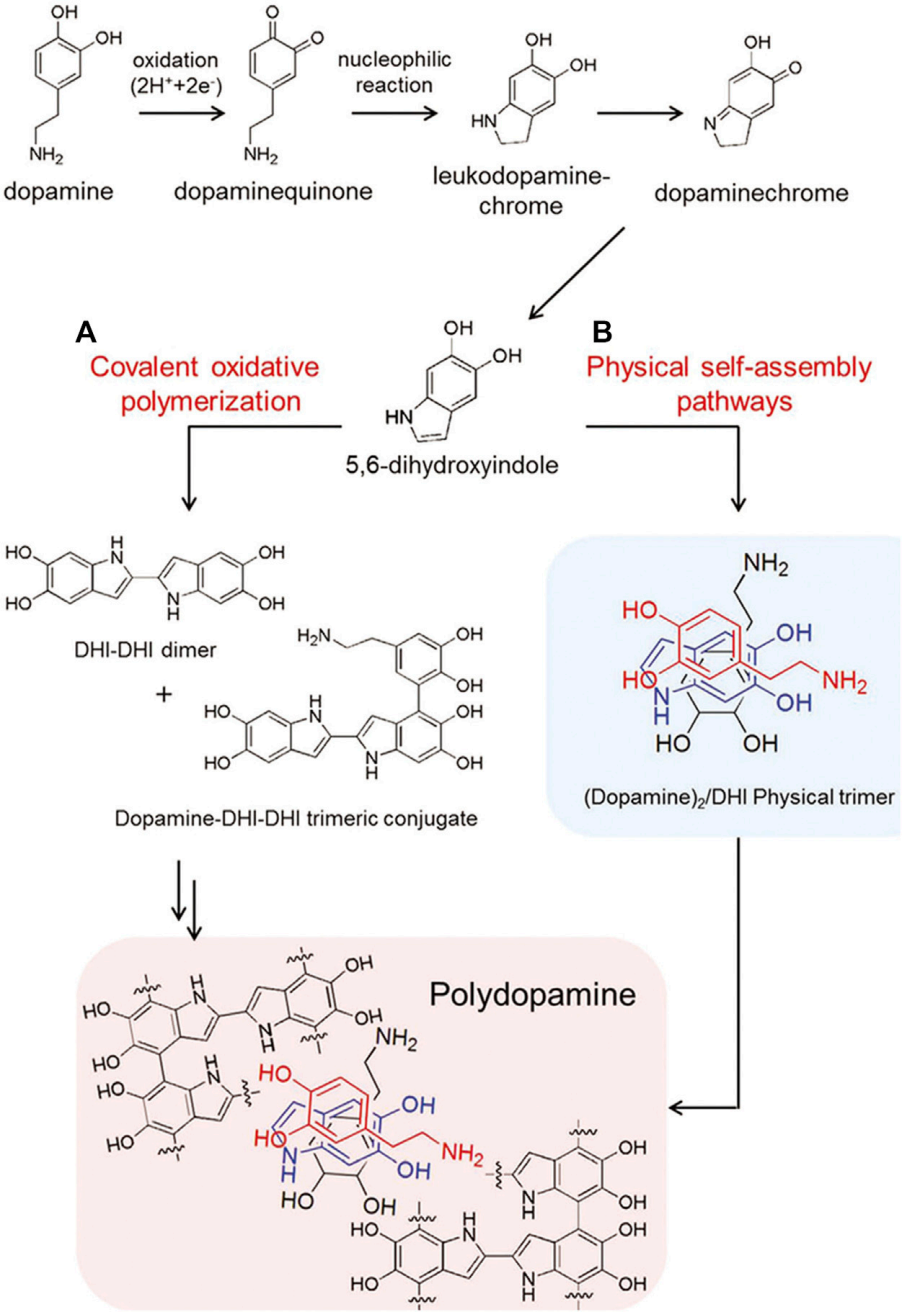
Schematic illustration for synthesis of PDA *via* two pathways: **(A)** covalent oxidative polymerization and **(B)** physical self-assembly pathways (Hong et al., 2012). Copyright 2012, Wiley-VCH.

*Via* the polymerization of DA monomers, the PDA coating can be achieved on various surfaces, whose definite polymerization mechanism is still ambiguous. Until now, the most wide-accepted theory is that both covalent oxidative polymerization and physical self-assembly pathways contribute to DA polymerization (Hong et al., 2012). The catechol groups of DA can be easily oxidized in alkaline condition to form DA-benzoquinone, and subsequently, DA derivatives are formed *via* intramolecular cyclization of DA-benzoquinones, which will be further oxidized and thus produce 5, 6-dihydroxyindole. Finally, the formation of dark brown PDA is achieved through cross-linking following intermolecular and intramolecular rearrangement (Figure 3).

In addition, the exact mechanism of the robust adhesion capability of PDA remains complicated. Nonetheless, it’s widely accepted that the binding energy between PDA coating and substrates mostly comes from covalent and noncovalent binding, while the multifunctional groups of PDA coating, including catechol, amino, carboxylic acid, indole units, and quinone groups, endow the modified substrates with mussel-mimicking multifunctional capacities (Tang et al., 2021).

### Property and Function of PDA-Modified Surfaces

**FIGURE 4:**
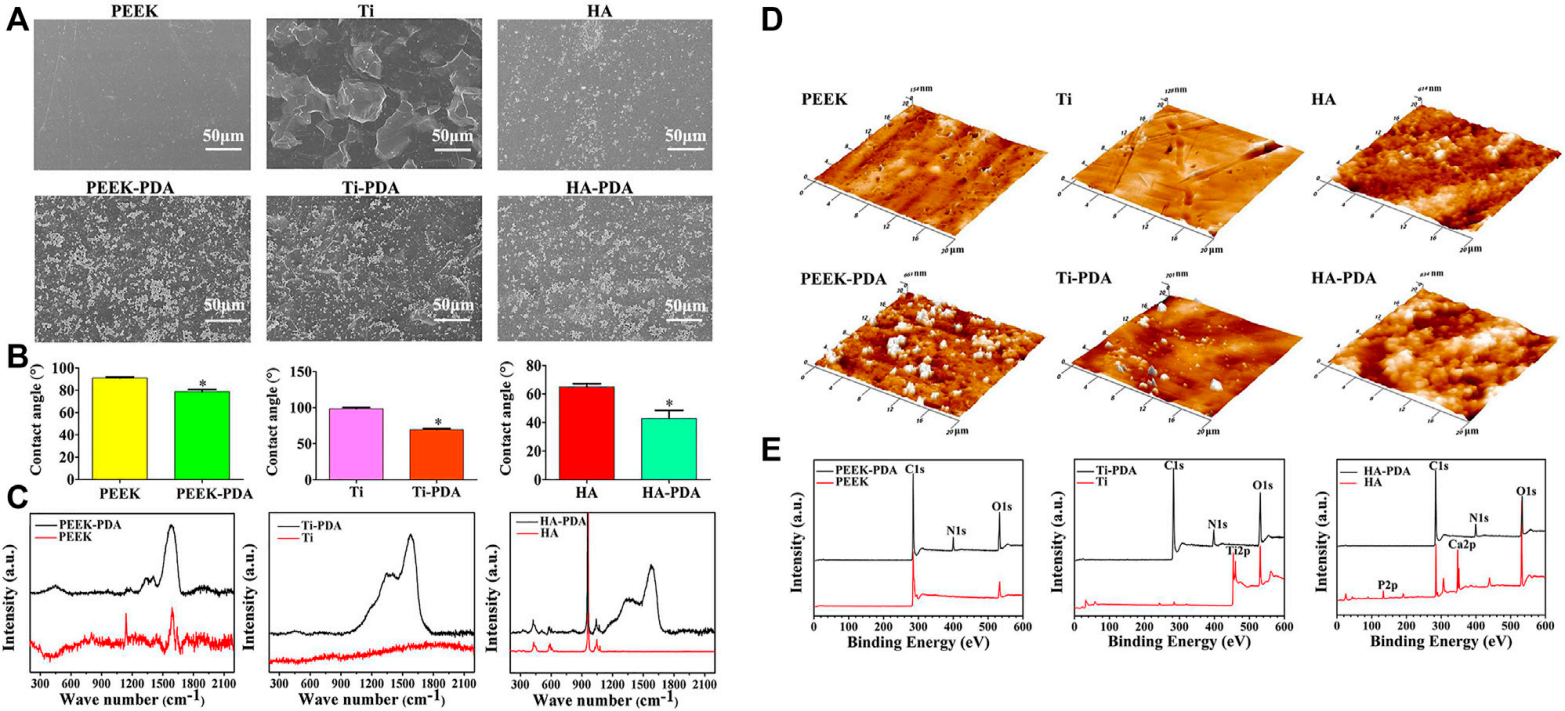
Changes of surface physiochemical property after PDA modification on different biomaterials. Changes of surface morphology observed by **(A)** SEM and **(D)** AFM images; **(B)** Changes of surface wettability; Changes of chemical conponents detected by **(C)** Raman spectra and **(E)** XPS analysis, the characteristic peaks of PDA can be observed (Wang H. et al., 2019). Copyright 2019, American Chemical Society.

**FIGURE 5:**
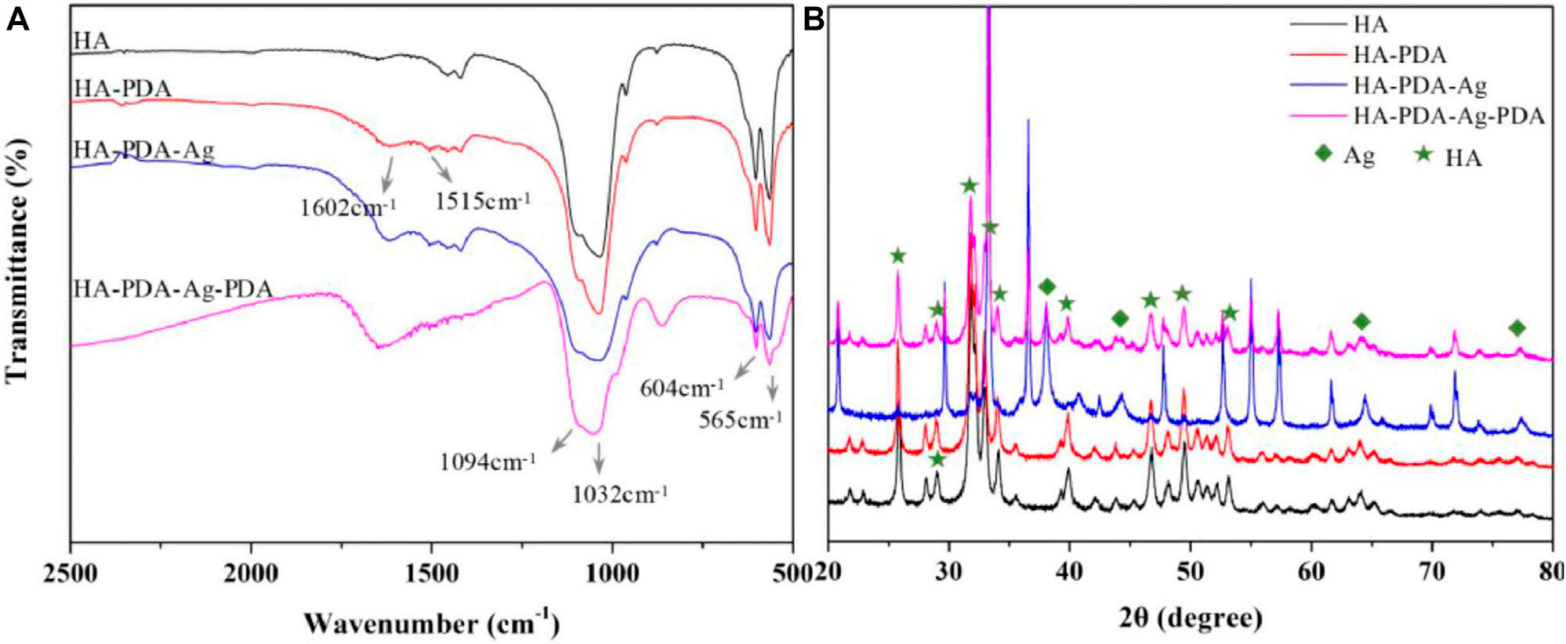
After PDA modification and Ag decoration, changes of chemical components on HA substrates are detected by **(A)** FTIR, and the characteristic peaks are identified. The characteristic peaks of PDA (1602 cm^-1^ and 1515 cm^-1^) can be observed. Changes of crystalline structures are detected by **(B)** XRD analysis, and the crystalline structures stay unchanged after PDA modification, where the quadrangles represent peaks of Ag and the pentagrams represent peaks of HA. (Zhang et al., 2021). Copyright 2021, Elsevier.

After PDA modification, there are distinct differences in surface physiochemical property (Figure 4). Under scanning electron microscope (SEM) and atomic force microscope (AFM), the granular PDA aggregates can be observed formed and spread uniformly (Figures 4A,D), with increased surface roughness (Wang H. et al., 2019; Ma et al., 2019; Yin et al., 2019). Meanwhile, the hydrophilicity is also improved (Figure 4B), which has been proved able to enhance cell adhesion, proliferation and differentiation (Wang P. et al., 2021). As for chemical characterization, many chemical characterization techniques like Fourier transform infrared spectroscopy (FTIR) (Figure 5A), X-ray photoelectron spectroscopy (XPS) (Figure 4E) and Raman spectra analysis (Figure 4C) are used to prove the formation of PDA coating *via* changes of surface chemical components, where the characteristic peaks of PDA can be observed in different spectra. But the crystalline structures stay unchanged, demonstrated by X-ray diffraction (XRD) (Figure 5B) (Sun et al., 2013; Li L. et al., 2019; Yin et al., 2019). Notably, the catecholamine moieties of PDA coating can interact with Ca^2+^ ions, and thus promote biomimetic mineralization of hydroxyapatite (HA) (Ghorbani et al., 2019). Furthermore, when applied on biodegradable materials, the PDA coating can help improve corrosion resistance (Bi et al., 2019).

**FIGURE 6:**
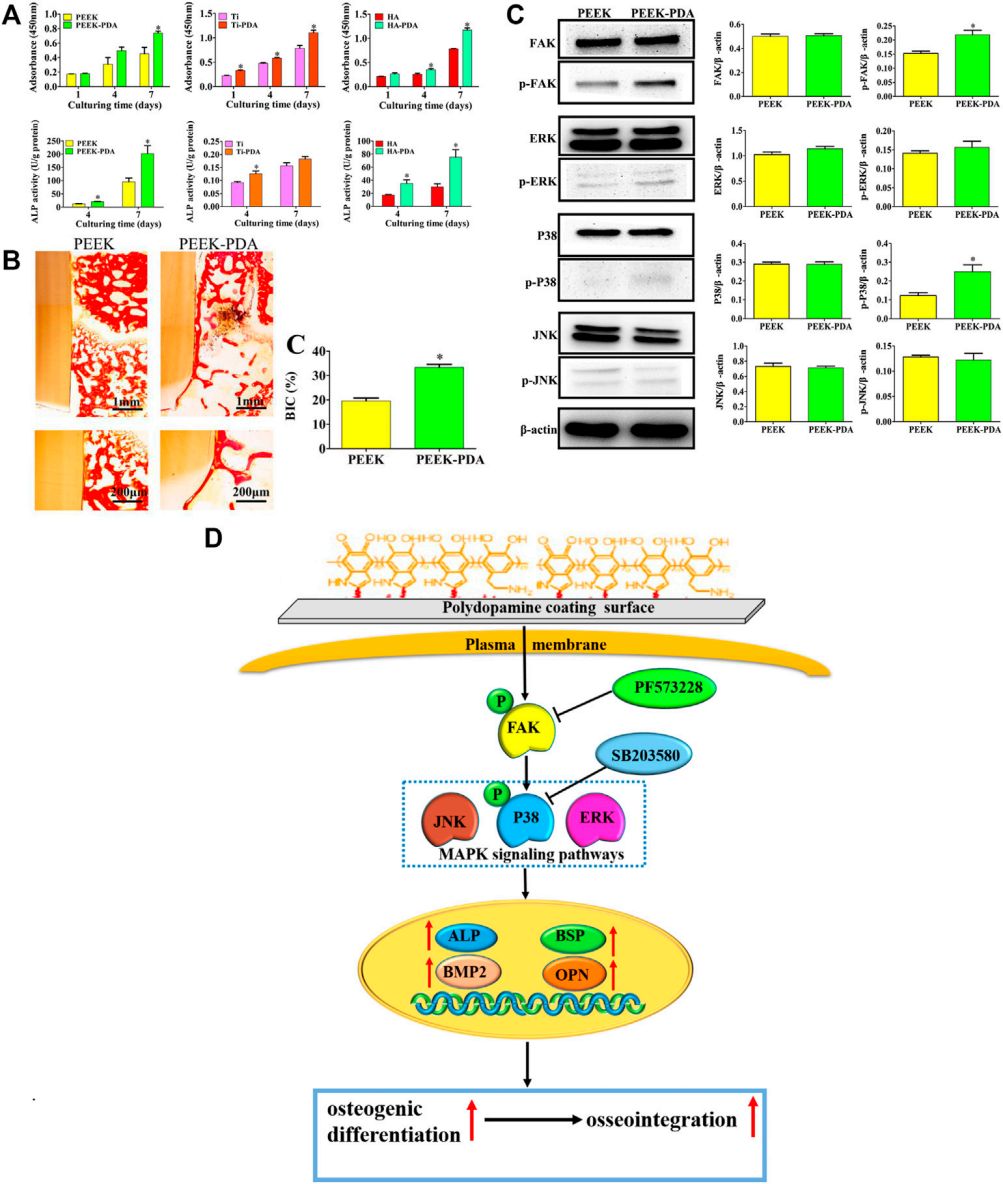
The osteogenic activity and mechanism of PDA-coated Ti, PEEK and HA substrates. **(A)** The enhancement of cell proliferation and osteogenic differentiation of BMSCs on PDA-coated substrates. **(B)** The enhanced osseointegration of PDA-modified PEEK implants *via* histomorphometry analysis. **(C)** Western blot experiments and quantitative analysis of the expression of FAK, p-FAK, and MAPK signaling pathways proteins of BMSCs after culturing for 48 h. **(D)** Schematic diagram for the mechanism of enhanced osteogenic differentiation and osseointegration of PDA coating. (*p < 0.05) (Wang H. et al., 2019). Copyright 2019, American Chemical Society.

The independent PDA coating without introducing other chemical composition has been proved beneficial for osteogenesis by plenty of researches, where the PDA coating itself has exhibited multiple biofunctions, including enhancement of cell adhesion, proliferation and osteogenic differentiation (Sun et al., 2013; Li L. et al., 2019; Wang H. et al., 2019; Yin et al., 2019; Zhong et al., 2020; Wang P. et al., 2021), as well as immunomodulation (Liu et al., 2021) and biomineralization (Ghorbani et al., 2019; Kaushik et al., 2020). Liu et al. (Liu et al., 2021) found that the PDA coating enabled early and durable influx of anti-inflammatory (M2) subtype of macrophages, which can in turn promote bone marrow mesenchymal stem cells (BMSCs) recruitment, osteogenic differentiation and new bone formation in alveolar defect. Hui Wang et al. (2019) demonstrated that the PDA coating on titanium (Ti), polyetheretherketone (PEEK) and HA could regulate p38 and focal adhesion kinase (FAK) signaling pathways to enhance adhesion, proliferation and osteogenic differentiation of BMSCs, and thus accelerated osseointegration in SD rat models (Figure 6). Therefore, PDA coating itself seems a universal strategy to modify biomaterial surfaces for enhanced new bone formation.

## PDA-Mediated Components for Multilayered Coatings on Biomaterials

Due to multifunctional groups, the PDA coating is able to serve as a general platform for further treatments to form multilayered coatings, where different techniques have been applied, such as dip-coating, spray-coating, spin-coating, hydrothermal methods and so on. Through these coating techniques, different chemical components can be immobilized on PDA-coated substrates, and thus endow them with multiple biofunctions. Meanwhile, the PDA coating largely enhances the adhesive property of modified substrates, and in turn enhances the binding strength of deposited chemical components. In this part, we summarize different components involved in PDA-based multilayered coatings and their biofunction, such as metallic components, inorganic particles, polymeric scaffolds, proteins, peptides, drugs and so on, which can further provide inspiration for fabrication of multifunctional scaffolds for better bone formation.

### PDA-Mediated Coatings Containing Metallic Components

During surface modification, the metallic components are often applied in the form of elementary substance or oxide, while under the *in vivo* condition, the released metallic ions, as the functional components, play a key role in tissue repair. However, if local concentration of metallic ions is too high, the surrounding tissues will be damaged by the overdosage of potential cytotoxicity, which is caused by the rapid release of metallic ions and may have a negative effect on bone formation (Kirkland et al., 2010; Xie et al., 2019; Xiong et al., 2022). Therefore, it is important to further apply ion carriers with favorable releasing property to achieve a sustained release of metallic ions, which can be realized by introduction of external components to the PDA-based multilayered coating *via* layer-by-layer process. There are several metallic ions that are commonly introduced to PDA-based multilayered coatings, including silver ion (Ag^+^), strontium ion (Sr^2+^), magnesium ion (Mg^2+^), zinc ion (Zn^2+^) and copper ion (Cu^2+^) (Table 1).

**TABLE 1:** Functional metallic ions applied in PDA-mediated multifunctional coatings.

Ions	Techniques	Advantages	References
Ag^+^	Dip-coating and subsequent reduction	Antibiosis	Tejido-Rastrilla et al. (2018), Han et al. (2019), Tejido-Rastrilla et al. (2019), Xie et al. (2019), Deng et al. (2020b), Albashari et al. (2021)
Sr^2+^	Hydrothermal; Electrochemistry; Dip-coating	Improving bone remodeling; Angiogenesis	Wei et al. (2017), Shengnan Wang et al. (2019), Cheng et al. (2021), Yiting Sun et al. (2021), Bingbing Wang et al. (2021)
Mg^2+^	Dip-coating	Enhanced new bone formation	Ma et al. (2020)
Zn^2+^	Dip-coating	Improving bone remodeling; Antiinflammation	Peng et al. (2021)
Cu^2+^	Dip-coating; Spin-coating	Antibiosis; Less toxicity	Mingjun Li et al. (2021), Lei Wang et al. (2021), Yan et al. (2021)

Ag^+^ is an agent with broad-spectrum antimicrobial property, which can disrupt bacterial metabolism *via* increasing bacterial membrane permeability and inducing intracellular oxidation (Wang G. et al., 2017). Through direct immersion in silver nitrate solution and subsequent reduction to Ag under ultraviolet irradiation, and thus Ag nanoparticles can be directly decorated on PDA-modified substrates (Tejido-Rastrilla et al., 2018; Han et al., 2019; Tejido-Rastrilla et al., 2019; Xie et al., 2019; Deng et al., 2020b; Albashari et al., 2021). Deng (Deng et al., 2020b) developed a “PDA−Ag−PDA” sandwich structure on PEEK substrates *via* layer-by-layer process, which exhibited bacteria-triggered pH-responsive ion-releasing behavior, as well as biocompatibility and osteogenic activity.

Sr^2+^ is a natural component in bone tissue, which is able to maintain the balance between osteoclasts and osteoblasts and improve bone remodeling (Ullah et al., 2020; Praharaj et al., 2022). It is also crucial for vascular development (Zhao et al., 2018). When combined with HA, the osteogenic activity will be synergistically enhanced (Wei et al., 2017; Wang S. et al., 2019; Wang B. et al., 2021; Sun Y. et al., 2021). Sun et al. (Sun Y. et al., 2021) firstly fabricated PDA coating as intermediate layer on Ti substrate and further constructed a Sr-substituted apatite coating, which was able to regulate PI3K/AKT and FAK/MAPK signaling pathways, and in turn promote osteogenesis and angiogenesis.

Mg^2+^, Zn^2+^ and Cu^2+^ ions are also essential for human body with physiological activity. Zn^2+^ is able to inhibit bone resorption and promote osteogenesis, and exhibits anti-inflammatory property (Shen et al., 2019). Mg^2+^ can also enhance new bone formation (Nieves, 2014). Cu^2+^ exhibits broad-spectrum antibacterial property and is less toxic than Ag (Slavin et al., 2017). The dip-coating technique is effective for introducing these ions on PDA coating to successfully fabricate multilayered coatings (Ma et al., 2020; Li M. et al., 2021; Wang L. et al., 2021; Peng et al., 2021; Yan et al., 2021). Ma (Ma et al., 2020) immobilized Mg^2+^ on PDA-modified porous tantalum (Ta) scaffolds to improve its vascularization and osteogenesis activity. Peng (Peng et al., 2021) developed a Zn-contained PDA-based multilayered coating on Mg alloy, which was able to inhibit bacterial infection and promote bone formation. Wang (Wang L. et al., 2021) loaded Cu^2+^ on PDA-modified PEEK substrate and demonstrated the contribution of ion release for enhanced antibacterial capacity and osteogenic activity.

Besides, different metallic oxides are also able to deposit on PDA coating for multi-functionalization, including but not limited to TiO_2_ (Xian et al., 2020), Fe_3_O_4_ (Huang et al., 2020), ZnO (Wang Z. et al., 2021), Cu_2_O/CuO (Xu et al., 2020; Yan et al., 2020), where the biofunction they are going to exhibit mainly depends on the released ions under *in vivo* environment, like antibacterial property and osteogenic activity (Table 2). Yan et al. (Yan et al., 2020) developed a multifunctional coating on PEEK substrate with osteogenic and antibacterial property *via* combination of PDA intermediate coating, copper oxide microspheres, Ag nanoparticles and silk fibroin. Under low PH environment, this multilayered coating could liberate Ag^+^ and Cu^2+^ and killed up to 99.99% planktonic bacteria, while under the physiological environment, the decreased leaked metal irons could lead to better osteogenesis.

**TABLE 2:** Metallic oxides applied in PDA-based multifunctional coatings.

Components	Techniques	Advantages	References
TiO_2_	Hydrothermal	Biocompatibility; Antibiosis; Osteogenesis	Xian et al. (2020)
Fe_3_O_4_	Dip-coating	Biocompatibility; magnetism	Huang et al. (2020)
ZnO	Dip-coating	Antibiosis	Zheng Wang et al. (2021)
Cu_2_O/CuO	Spin-coating; Dip-coating	Antibiosis	Xu et al. (2020), Yan et al. (2020)
Al_2_O_3_	Electrochemistry	Corrosion resistance	Xiyuan Wang et al. (2019)

### PDA-Mediated Coatings Containing Inorganic Particles

Bioceramic particles are the main inorganic components of PDA-based multilayered coating with osteogenic property, good compression and corrosion resistance (Turnbull et al., 2018). The inorganic calcium phosphates are the most frequently used bioceramics, among which hydroxyapatite [HA, Ca_10_(PO4)_6_(OH)_2_] and β-tricalcium phosphate [β-TCP, Ca_3_(PO_4_)_2_] have been most widely applied in orthopedic and dental fields for enhanced bone formation. Notably, the HA particles deposited on PDA-modified surfaces can be achieved *via* PDA-mediated biomineralization (Hasani-Sadrabadi et al., 2019; Park et al., 2021). Bi Wang et al. (2017) developed a multilayered coating *via* PDA-assisted assembly of HA and Ag nanoparticles on Mg substrates, which exhibited enhanced corrosion resistance, antibacterial property and biocompatibility towards osteoblasts.

Silica-based bioceramic particles such as silica (SiO_2_), bioactive glass (BG, CaO–SiO_2_–P_2_O_5_ composition) and calcium silicate ceramics, have also received significant interest as promising biomaterials for bone regeneration, whose ionic breakdown products exhibit osteoconductive property (Ho and Ding, 2015; Turnbull et al., 2018; Nikolova and Chavali, 2019; Yuan et al., 2022). Ho and Ding (Ho and Ding, 2015) developed a novel SiO_2_/PDA hybrid coating *via* dip-coating technique, which improved biological responses of MG63 osteoblasts, including cell adhesion, proliferation, osteogenic differentiation and mineralization. Furthermore, Ding et al. (2020) loaded mesoporous silica nanoparticles (MSNs) with Ag particles to realize antibacterial property on PDA-modified Ti surface, which enhanced bone formation at implant-tissue interface.

However, the binding strength of bioceramic nanoparticles to PDA intermediate layer is not so satisfying just with covalent and noncovalent interaction, and the coating will face the risk of destruction during application. Thus, this type of coating should be applied to non-bearing bone defects.

In addition, graphene oxide (GO), one of graphene derivatives, is another important inorganic component, and shows good biocompatibility and osteogenic activity (Jia et al., 2016). Usually, GO can be immobilized on biomaterials’ surfaces by immersion and the following decoration can be achieved *via* layer-by-layer process, where the initial PDA modification of substances can largely improve adhesion of GO *via* π–π stacking and electrostatic interplay (Han et al., 2018; Deng et al., 2020a; Wang S. et al., 2020; Xu K. et al., 2021). Kui Xu et al. (2021) prepared multilayered PDA/GO/Col I nanofilms on Ti substrates *via* layer-by-layer self-assembly, which displayed excellent biocompatibility and controllable release of bioactive substances, as well as enhanced protein adsorption and osteogenic differentiation.

### PDA-Mediated Coatings Containing Polymeric Components

The polymeric components applied on PDA-based multilayered coatings include natural and synthetic polymers and often exhibit biodegradability. Natural polymers, such as silk (Jia et al., 2019; Asadi et al., 2021; Xu C. et al., 2021; Sang et al., 2021), chitosan (Li X. et al., 2019; Xie et al., 2019), gelatin (Dinh et al., 2018; Han et al., 2018; Su et al., 2022), alginate (Xu et al., 2020) and so on, exhibit favorable ductility, biocompatibility and biodegradability, and often contain bioactive molecules, which can be beneficial for new bone formation (Turnbull et al., 2018). Synthetic polymers, including poly(lactic-co-glycolic acid) (PLGA), poly(lactic acid) (PLA), poly(ethylene glycol) (PEG), poly(caprolactone) (PCL), poly(glycolic acid) (PGA) and so on, can be fabricated with customized shape, pore size, porosity, degradation rate and mechanical property to match the individual demands (Turnbull et al., 2018; Sharma et al., 2022). However, synthetic polymers are usually used as biomedical scaffolds while natural polymers actually serve as components of multilayered coatings in most cases.

Due to the outstanding adhesiveness and high reactivity towards amine, thiol and quinone functional groups, PDA-modified substrates can be decorated by polymers with functional groups *via* covalent attachment to achieve better stability (Jia et al., 2019; Xu C. et al., 2021). Besides osteogenic activity, the polymer-loaded multilayers can serve as a localized depot and delivery platform for biomolecules and drugs due to their structural property, and thus exhibit property of antibacterial, antiinflammation, as well as enhanced osteogenic activity (Jia et al., 2019; Xie et al., 2019; Xu et al., 2020; Xu C. et al., 2021; Sang et al., 2021). Jia et al. (Jia et al., 2019) applied a simple combination strategy to achieve a multilayered silk/AgNP hierarchical biofunctionalized structure on PDA-modified 3D-printed Ti6Al4V scaffold, which could effectively reduce bacterial adherence, and enhance cell proliferation and osteogenic differentiation of MC3T3-E1 cells.

Notably, there also exist disadvantages of natural polymers, such as weak biomechanics, low stiffness, poor processability, inappropriate degradation rate, lack of surface specificity and high price (Nikolova and Chavali, 2019). Additionally, some natural polymers are possible to induce inflammation of hosts (Zhang et al., 2019).

### PDA-Mediated Coatings Containing Proteins and Peptides

#### PDA-Mediated Protein-Contained Coatings

Bone morphogenetic protein-2 (BMP-2) is one of the most effective members of the BMP family that can enhance *in vivo* new bone formation, and thus has attracted much attention in research and clinic fields (Lee et al., 2016; Jiang et al., 2017; Zhao et al., 2017; Lee et al., 2018; Han et al., 2019; Wan et al., 2019; Bedair et al., 2020). Lee et al. (Lee et al., 2016) attached human bone BMP-2 onto 3D-printed PCL scaffold *via* PDA intermediate layer, and achieved a sustained release pattern to promote osteoconductivity. However, BMP-2 is associated with several adverse effects like bone overgrowth and immune reactions, which should be carefully considered beforehand and thus limit its application (Pan et al., 2016).

Another common protein for bone regeneration is type I collagen (Col I). Col I is an indispensable components of human bone tissue, which has been proved able to promote osteogenic differentiation and biomineralization of extracellular matrix (ECM) (Rother et al., 2015; Wegst et al., 2015; Qian et al., 2019; Guo et al., 2020; Xu K. et al., 2021; Zhou et al., 2021). Guo et al. (Guo et al., 2020) fabricated a multifunctional composite coating *via* a two-step chemical method to load PDA, dicalcium phosphate and COL I sequentially. The multilayered coating improved the corrosion resistance of Mg alloys, exhibited similar composition to natural bone, and obviously enhanced osteogenic differentiation of MC3T3-E1 cells.

VEGF and bFGF are growth factors with similar function, which can effectively stimulate angiogenesis of vascular endothelial cells and promote osteogenic differentiation of osteoblasts (Wu et al., 2020; Li J. et al., 2021). Godoy-Gallardo et al. (2020) reported a PDA-based multilayered coating on PCL/HA scaffold for bone regeneration, where BMP-2 and VEGF were loaded. After evaluation, a synergistic enhancement of osteogenic gene expression could be observed, which is greater than scaffolds loaded with individual BMP-2 and VEGF. Besides, during fabrication of PDA-based multilayered coatings, other components can also be combined with VEGF and bFGF for extended multifunctionalization, like metallic ions and bioceramics (Albashari et al., 2021; Li J. et al., 2021). Albashari et al. (Albashari et al., 2021) designed a bFGF cross-linked Ag coating *via* PDA and heparin intermediate layer on Ti substrates, which showed satisfying osteogenic differentiation, anti-inflammatory and antibacterial property.

Despite a relatively less attention, other proteins, such as adiponectin (APN), insulin-like growth factor-1 (IGF-1) and lactoferrin, also have been applied to bone regeneration *via* PDA intermediate layer. Deng et al. (Deng et al., 2020a) applied APN on GO/PDA complex to create a multilayered coating on PEEK substrates, where the APN component was proved by *in vitro* and *in vivo* experiments to enhance biocompatibility and osteogenic activity. IGF-1 has been proved able to promote osteoblast differentiation through the mTOR signaling pathway (Feng et al., 2014; Qiu et al., 2018). Several studies have combined BMP-2 and IGF-1 to achieve a better performance of osteogenic differentiation on PDA-modified substrates, which has been proved better than independent release of BMP-2 or IGF-1 (Chen et al., 2009; Zhang et al., 2017; Wan et al., 2019). In addition, Shen et al. (Shen et al., 2018) used PDA-assisted biomineralization and layer-by-layer spin-coating technique to develop a multilayered structure containing HA and lactoferrin on the Ti substrate. The results proved the toxicity to osteoblasts of high-concentration lactoferrin, which could be improved *via* HA deposition on PDA intermediate layer. Furthermore, the lactoferrin has been proved to prevent NF-kB activation and inhibit pro-inflammatory cytokine synthesis, which will exhibit anti-inflammatory activity (Ward et al., 2005). In addition, lactoferrin can significantly stimulate a variety of cells differentiation toward osteoblasts, inhibit the formation and activity of osteoclasts, and further regulate bone homeostasis (Shen et al., 2018). Therefore, the lactoferrin could promote osteogenesis *via* the combination of its direct osteogenic activity and indirect effect of regulated immune response.

#### PDA-Mediated Peptide-contained Coatings on Implants

Compared to full-length counterparts, peptides have better resistance and can maintain their activity after harsh treatment (Firestone and Chen, 2010). They are also less expensive with better stability and easier accessibility (Zhang et al., 2020). BMP-derived peptides, collagen-derived peptides, osteogenic growth peptides and arginine-glycine-aspartic acid (RGD) peptide family are commonly used peptides to form PDA-based multilayered coatings (Sun et al., 2013; Pan et al., 2016; Wang et al., 2016; Bai et al., 2020; Zhang et al., 2020; Zhou et al., 2020; Yang et al., 2021). Wang et al. (Wang et al., 2016) developed a biomimetic process to prepare functional substrate surfaces with collagen-derived peptides and osteogenic growth peptides *via* PDA intermediate coating and found this multifunctional coating could promote osteogenic differentiation and biomineralization. Besides, antimicrobial peptides, such as nisin and KR-12 (Wang N. et al., 2020; Meng et al., 2020), also have attracted extensive interests because they are rarely toxic and have broad-spectrum antibacterial property with less risk of bacterial resistance (Zasloff, 2002; Brogden, 2005). Meng et al. (Meng et al., 2020) immobilized KR-12 on PDA-modified PEEK substrate, where the as-prepared surface showed better antibacterial activity against *staphylococcus aureus* and promoted osteogenic activity both *in vitro* and *in vivo*.

### PDA-Mediated Drug-Loaded Coatings

Drugs loaded on PDA-based multilayered coatings can be classified according to their functions, such as antibacterial, immunomodulation, homeostasis regulation, angiogenesis, antitumor and anti-tuberculosis (Table 3). Due to the PDA-assisted sustained release of drugs around the scaffolds or implants, the local drug concentration could be maintained at a favorable level, which will benefit the *in vivo* functionalization.

**TABLE 3:** Drugs applied in PDA-based multifunctional coatings.

Function	Drugs	References
Antibiotics	Vancomycin; Gentamicin; Chlorhexidine; Metronidazole; Moxifloxacin hydrochloride; Daptomycin; Citrate	Daud et al. (2018), Han et al. (2018), Zhang et al. (2018), Zeng et al. (2020), Zhang et al. (2020), Gao et al. (2021), An'an Sun et al. (2021), Yan et al. (2021)
Immunomodulation	Dexamethasone; Aspirin; Chondroitin; Sulfate; Salidroside	Tejido-Rastrilla et al. (2018), Ya-Min Li et al. (2019), Xu et al. (2019), Wei et al. (2020), Rozanc et al. (2021), Xiao et al. (2021), Yi et al. (2022)
Angiogenesis	Citrate	Yan et al. (2021)
Homeostasis regulation	Insulin; Metformin	Yang et al. (2019), Wei et al. (2021)
Antitumor	Doxorubicin	Shao et al. (2021)
Antituberculosis	Isoniazid; Rifampicin	Hua et al. (2022)

Antibiotics, including vancomycin (Han et al., 2018; Zhang et al., 2018; Zhang et al., 2020), gentamicin (Sun A. et al., 2021), chlorhexidine (Daud et al., 2018), metronidazole (Zhang et al., 2020), moxifloxacin hydrochloride (Gao et al., 2021), daptomycin (Zeng et al., 2020), as well as citrate (Yan et al., 2021), have been reported to form multilayered coatings on PDA-modified substrates, where the controlled drug release greatly enhanced the antibacterial property. Zhang et al. (Zhang et al., 2018) fabricated a PDA-based multilayered coating to load vancomycin and heparin on 3D-printed Ti6Al4V scaffolds for customized reconstruction of intricate bone defects, which possessed satisfying antibacterial, osteogenic and anticlotting property due to the high loading capacity and sustainable drug release ability.

Drugs for immunomodulation include dexamethasone (Tejido-Rastrilla et al., 2018; Xu et al., 2019; Rozanc et al., 2021; Xiao et al., 2021), chondroitin sulfate (Li Y.-M. et al., 2019), salidroside (Yi et al., 2022), aspirin (Wei et al., 2020) and so on. Among different antiinflammation drugs, dexamethasone can actually alleviate all levels of inflammation and is commonly used to treat various inflammatory diseases (Rozanc et al., 2021). Xu et al. (Xu et al., 2019) developed a PDA-mediated dexamethasone plus minocycline-loaded liposomes on PEEK substrates. The multifunctional coating can effectively modulate cell inflammatory response and discourage bacterial colonization, where the osteogenic activity could be synergistically enhanced both *in vitro* and *in vivo*.

Insulin and metformin are drugs for patients with hyperglycemia to regulate energy homeostasis, which also exhibit potential in bone tissue engineering. Wei et al. (Wei et al., 2021) found that the PDA-mediated insulin-releasing PCL composite scaffold could improve osteochondral repair after implanted into rabbit osteochondral defects. Yang et al. (Yang et al., 2019) prepared a PDA-templated HA scaffold and further demonstrated that the introduction of metformin could increase the cell viability of human periodontal ligament stem cells through the AMPK/mTOR signaling pathway and thus enhanced their osteogenic differentiation.

Besides antibacterial property, citrate has also been reported to induce angiogenesis (Binu et al., 2013). Yan et al. (Yan et al., 2021) utilized the co-deposition of PDA and Cu-citrate nanoclusters to form a pH-responsive coating on PEEK substrates, which could release Cu ions and citrate at lower PH and exhibit outstanding antibacterial and anti-infection property, as well as enhanced vascular and bone formation.

The application of antitumor and anti-tuberculosis drugs to assist osteogenesis also have been explored in specific situations. Shao et al. (Shao et al., 2021) loaded doxorubicin on Mg alloy *via* PDA intermediate coating for bone reconstruction after resection of primary malignant bone tumors. The multilayered coating showed outstanding photothermal property and sustained doxorubicin release for tumor inhibition, where the osteogenic activity was also enhanced. Hua et al. (Hua et al., 2022) developed a PDA-modified 3D-printed Ta scaffold to load isoniazid and rifampicin for the treatment of osteoarticular tuberculosis, which was demonstrated to achieve long-term drug release and bone regeneration at the same time.

The disadvantages of drug-loaded coating mainly lie in the rapid release rate and the risk of drug resistance, which will negatively affect the drug efficiency and inhibit the final bone formation. Therefore, it is significant to develop advanced drug delivery system on the PDA-based multilayered coating to achieve a controllable release rate, and overcome drug resistance, where nanoparticles become a promising strategy as a drug carrier (Lai et al., 2022). However, few clinical trials have been carried out to evaluate their safety and practical efficacy.

### PDA-Mediated Coatings Containing Other Components

Other components reported include small interfering ribose nucleic acid (siRNA), exosomes and ECM. Transfection of siRNA into stem cells can guide lineage specification of stem cells into different lineages, including osteogenesis (Cui et al., 2015). Shin et al. (Shin et al., 2016) used siRNA-lipidoid nanoparticles to decorate PLGA scaffolds *via* PDA intermediate coating and demonstrated its long-period stability, which could help silence an osteogenesis inhibitor gene and enhance osteogenic differentiation of human adipose-derived stem cells.

Exosomes are nanovesicles released by cell membrane fusion and are responsible for mediating local and cell-cell communication (Melo et al., 2015). Exosome-based regenerative therapy has advantages of low immunogenicity, high stability and homing effects (Li W. et al., 2018). Xing et al. (Xing et al., 2021) applied mesenchymal stem cells-derived exosomes to functionalize electrospun scaffolds with assistance of PDA intermediate layer. The modified scaffold was proved to promote adhesion, proliferation and osteogenic differentiation of preosteoblasts, as well as osseointegration in rat cranial bone defects.

ECM involves multiple macromolecules, which not only provides 3D structure to supports cells, but also exhibits stimulations of different important biological functions and eventually determines the fate of stem cells (Zhou et al., 2020). Wang et al. (Wang et al., 2018) used PDA intermediate layer to immobilize paraformaldehyde-treated ECM coating on Ti substrate, and found that the modified surface exhibited well-maintained structures and the bioactive components inside ECM could greatly promote osteogenic differentiation.

## PDA-Based Multilayered Coatings for Enhanced Bone Formation

Actually, in most researches, different components are often combined together to form multilayered coatings *via* PDA assistance, which can exhibit multiple functions. Since many different components show similar functions, a summarization of different functions can help to achieve a better understanding of PDA-based multilayered system. Therefore, in this part, besides osteogenic activity, we classify various multilayered coatings in terms of their functions and enhancement for bone formation, no matter which components are involved.

### Osteogenesis Activity of PDA-Based Multilayered Coatings

During bone regeneration progress, BMSCs recruitment is prerequisite to the healing process, which may come from periosteum, endosteum and blood circulation. After migration, BMSCs will infiltrate into hematoma, differentiate into osteogenic cells and participate in bone repair (Liu et al., 2021). Another vital factor for osteogenesis is the balance between bone resorption and bone formation, which are regulated by osteoclasts and osteoblasts (Durdan et al., 2022). It is better to upregulate activity of osteoblasts and inhibit osteoclasts to achieve faster bone formation during osteogenesis. Therefore, it is important to improve recruitment and differentiation of BMSCs and regulate bone remodeling.

**FIGURE 7:**
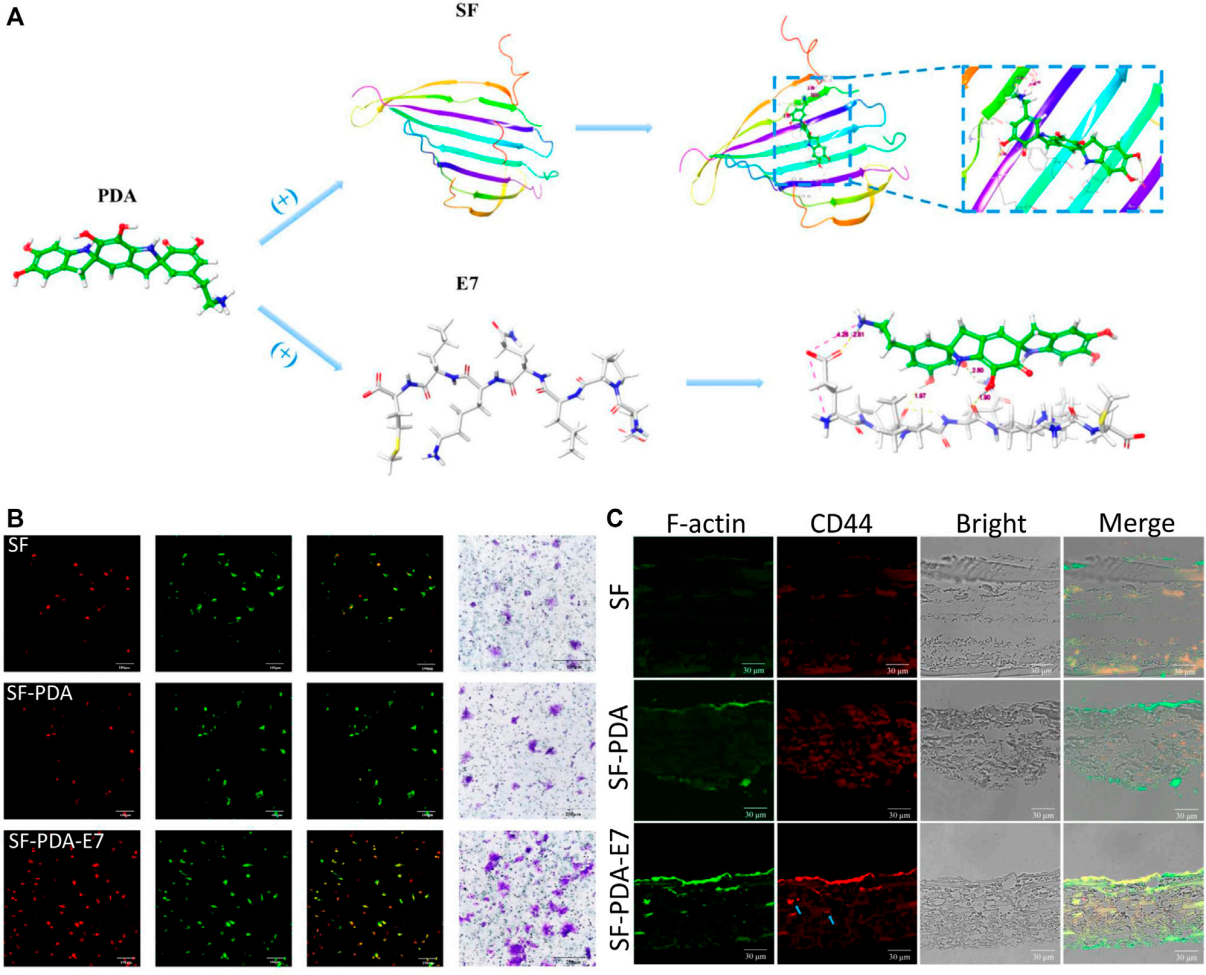
Recruitment of BMSCs induced by E7-loaded PDA-modified silk membrane. **(A)** Schematic illustration of the PDA-modified silk fibroin and PDA-mediated E7 peptide; **(B)** CD44 immunofluorescent staining of the *in vitro* recruited BMSCs after 3 days; **(C)** CD44 immunofluorescent staining of the *in vivo* recruited BMSCs after 7 days. Blue arrow: the BMSCs grew into the scaffolds (Wu et al., 2019). Copyright 2019, American Chemical Society.

Chemokines and different components with osteogenic activity are often used for better BMSCs recruitment and osteogenic differentiation. After loaded on the PDA-based multilayered coatings, chemokines can recruit more BMSCs to defected sites, and then bioactive components can promote osteogenic differentiation for enhanced bone formation (Kang et al., 2016; Pan et al., 2016; Wu et al., 2019). Wu et al. (Wu et al., 2019) used E7 peptide as a chemokine to decorate PDA-modified silk membrane, and the scaffolds exhibited high efficiency of BMSCs recruitment both *in vitro* and *in vivo*, which resulted from the regulation of the SDF-1α/CXCR4 axis, as well as the p38, ERK and Akt signal transduction pathways. The scaffolds also facilitated the osteogenic differentiation of BMSCs and enhanced new bone formation of the rat calvarial bone (Figure 7).

To modulate bone remodeling, a variety of bioactive components can be used to maintain the balance between osteoclasts and osteoblasts and enhance new bone formation, such as Sr^2+^, Zn^2+^, Mg^2+^, APN and IGF-1. After immobilized on PDA-based multilayered coatings, these components can directly inhibit osteoclast activity and upregulate bone formation. Cheng et al. (Cheng et al., 2021) incorporated Sr on Ta substrate *via* PDA intermediate layer and evaluated the osseointegration ability. The modified Ta scaffolds exhibited better osteogenic performance, where enhanced bone formation was observed in rat femur 12 months after implantation.

### Synergistic Multifunctions of PDA-Based Multilayered Coatings

Besides osteogenesis, PDA-based multilayered coating can exhibit plenty of other biofunction, such as immunomodulation, angiogenesis, antibiosis and antitumor property. These multifunctional coatings are designed to meet the demands of different application scenarios to ensure successful bone formation, which also explains why surface modification based on PDA coating is crucial for enhanced new bone formation (Table 4).

**TABLE 4:** Synergistic enhancement of multiple components and functions for new bone formation.

Functions	Substrates	PDA-mediated components	Techniques	Advantages	References
Osteogenesis	Silk membrane	Chemokine	Dip-coating	Enhanced BMSCs recruitment, osteogenic differentiation and new bone formation	Wu et al. (2019)
Osteogenesis	HA	Poly(L-lysine), BMP-2	Dip-coating	Sustainable drug releasing, enhanced osteogenic differentiation and bone formation of ectopic bone	Han et al. (2019)
Angiogenesis and osteogenesis	PCL/HA	BMP-2, VEGF.	Dip-coating	Synergistic enhancement of osteogenesis	Godoy-Gallardo et al. (2020)
Angiogenesis and osteogenesis	Ta	Sr	Dip-coating	Enhanced angiogenesis and osseointegration in rat femur	Cheng et al. (2021)
Antiinflamma-tion and osteogenesis	Ti	Osteogenic growth peptide	Dip-coating	Suppression of osteoclastogenesis and promotion of osteogenesis	Bai et al. (2020)
Antibiosis and osteogenesis	Zn	Sr-doped HA	Electroch-emical deposition	Corrosion resistance, antibacterial property and enhanced osteogenic activity	Bingbing Wang et al. (2021)
Antibiosis and osteogenesis	PEEK	Ag, CuO, silk fibroin	Spin-coating	PH-response, antibacterial property, enhanced osteogenic activity both *in vitro* and *in vivo*	Yan et al. (2020)
Antitumor and osteogenesis	Mg alloy	Doxorubicin	Dip-coating	Tumor suppression, photothermal property	Shao et al. (2021)

#### Immunomodulation Property

After biomaterials implanted, the immune system is activated and modulates the host environment, which will affect the following BMSCs recruitment and osteogenic differentiation (Vassey et al., 2020). Macrophages are the sentinels and regulators of the immune system, and the polarization of macrophages is closely related to bone remodeling. Researches have proved that the pro-inflammatory (M1) macrophage is related to bone resorption while the M2 macrophage is associated with tissue repair (Alhamdi et al., 2019; Bai et al., 2020). M1 macrophages secrete proinflammation cytokines to modulate cell phagocytosis, such as interleukin-12 (IL-12), tumor necrosis factor-*α* (TNF-*α*) and inducible nitric oxide synthase (iNOS). After the inflammation stage, M2 macrophages produce antiinflammation cytokines to promote cell proliferation, differentiation and bone modeling, such as transforming growth factor -*β* (TGF-*β*)*β*, interleukin-10 (IL-10) and interleukin-4 (IL-4). The balance between M1 and M2 macrophages exhibits a synergistic effect to enhance osteogenesis *via* secretion of growth factors, as well as regulation of immune response, BMSCs recruitment, osteogenesis and bone remodeling. As for some patients with specific diseases, like diabetes and osteoporosis, the balance between M1 and M2 macrophages is disrupted and bone resorption will be activated to negatively affect bone formation, which will interfere with bone remodeling and do harm to the repair of bone defects (Muñoz et al., 2020; Lee et al., 2021).

**FIGURE 8:**
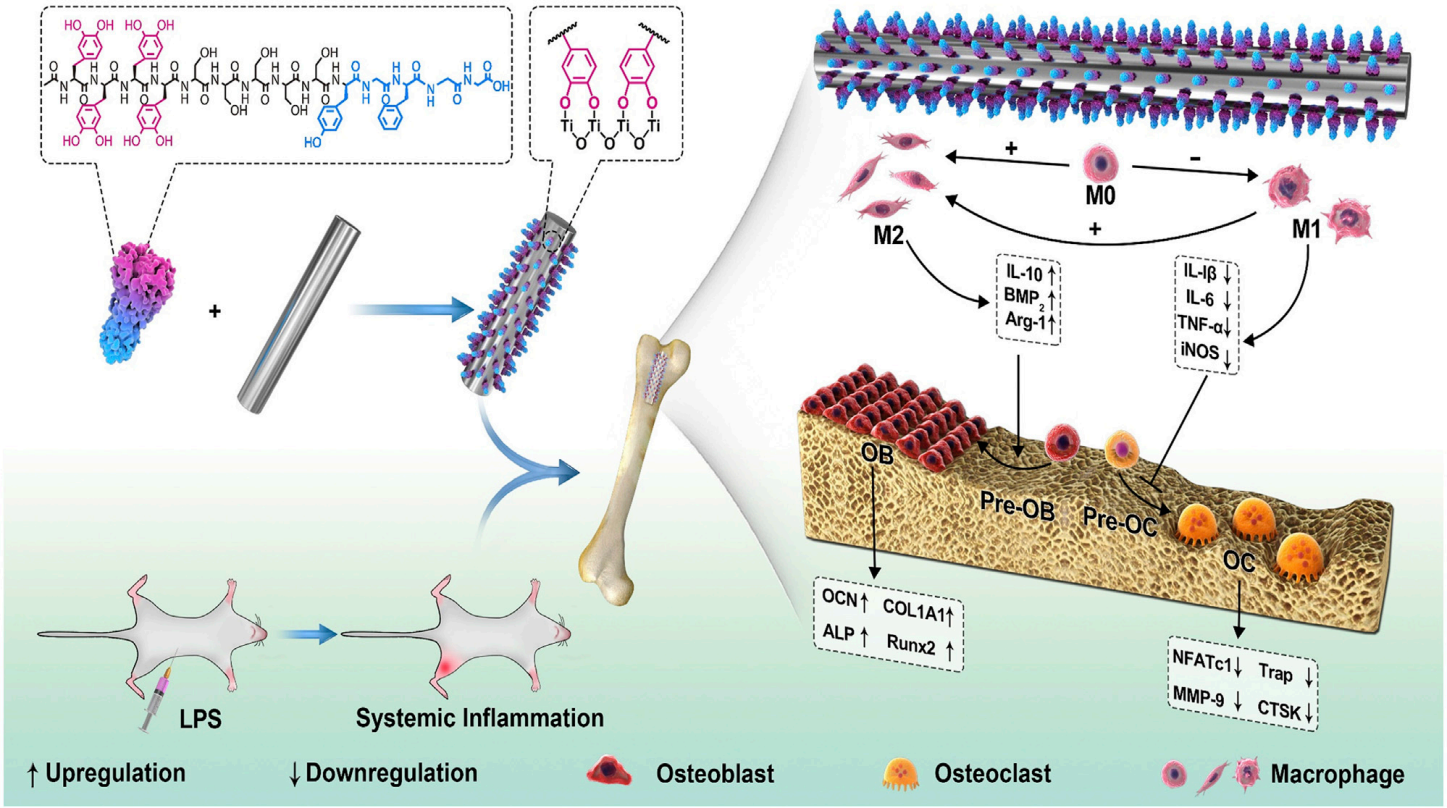
Schematic illustration for the effect of immunomodulation on osteogenesis *via* the PDA-based peptide-loaded coating. The peptide binds steadily to the Ti implant with the assistance of PDA. Under the LPS-induced inflammatory conditions, the biomimetic osteogenic peptide coating exhibited antiinflammatory property and changed the macrophages to the M2 phenotype, which in turn enhanced new bone formation (Bai et al., 2020). Copyright 2020, Elsevier.

Therefore, bioactive components with immunomodulation property are adoptable for regulation of bone remodeling, such as dexamethasone, chondroitin sulfate, salidroside, aspirin and some osteogenic growth peptides. Bai et al. (Bai et al., 2020) immobilized osteogenic growth peptides on PDA-coated Ti substrates to achieve a favorable bone immune microenvironment and promote interfacial osteogenesis. Due to satisfactory biocompatibility and mechanical properties, Ti and its alloys have been widely used in biomedical fields for hard tissue repair, but the bioinert characteristics can’t meet the demands of early osteointegration. Therefore, modifications of Ti and its alloys should be carried out for better biocompatibility, early osteointegration and abundant new bone formation. The results demonstrated that the modified Ti surface was able to promote osteogenesis and suppress osteoclastogenesis *via* inhibiting nuclear factor kappa-B (NF-κB) signalling pathway, where the M2 macrophages were upregulated and the bone-implant contact was increased by nearly three times (Figure 8).

#### Angiogenesis Property

Angiogenesis plays a vital role during the process of new bone formation, which helps transport nutrients to osteogenic cells and carry away metabolites simultaneously (Li J. et al., 2021). The blood vessels also provide structural support for bone development and mediate the recruitment and migration of BMSCs. Actually, the invasion of external blood vessels can largely affect the maintenance of cell viability. And the poor blood perfusion will cause cell death and treatment failure owing to the lack of nutrients and oxygen, as well as retained metabolites, which often occurs in the patients with large bone defects lacking sufficient blood supply (Grosso et al., 2017). Meanwhile, the angiogenesis can induce the differentiation of mesenchymal stem cells into osteoblasts and promote osteoid formation, where the enhanced osteoblast differentiation can in turn facilitate secretion of factors such as *β*-catenin and BMP-2 to promote the vascularization process. Therefore, osteogenesis is tightly connected with angiogenic process (Ding et al., 2021).

**FIGURE 9:**
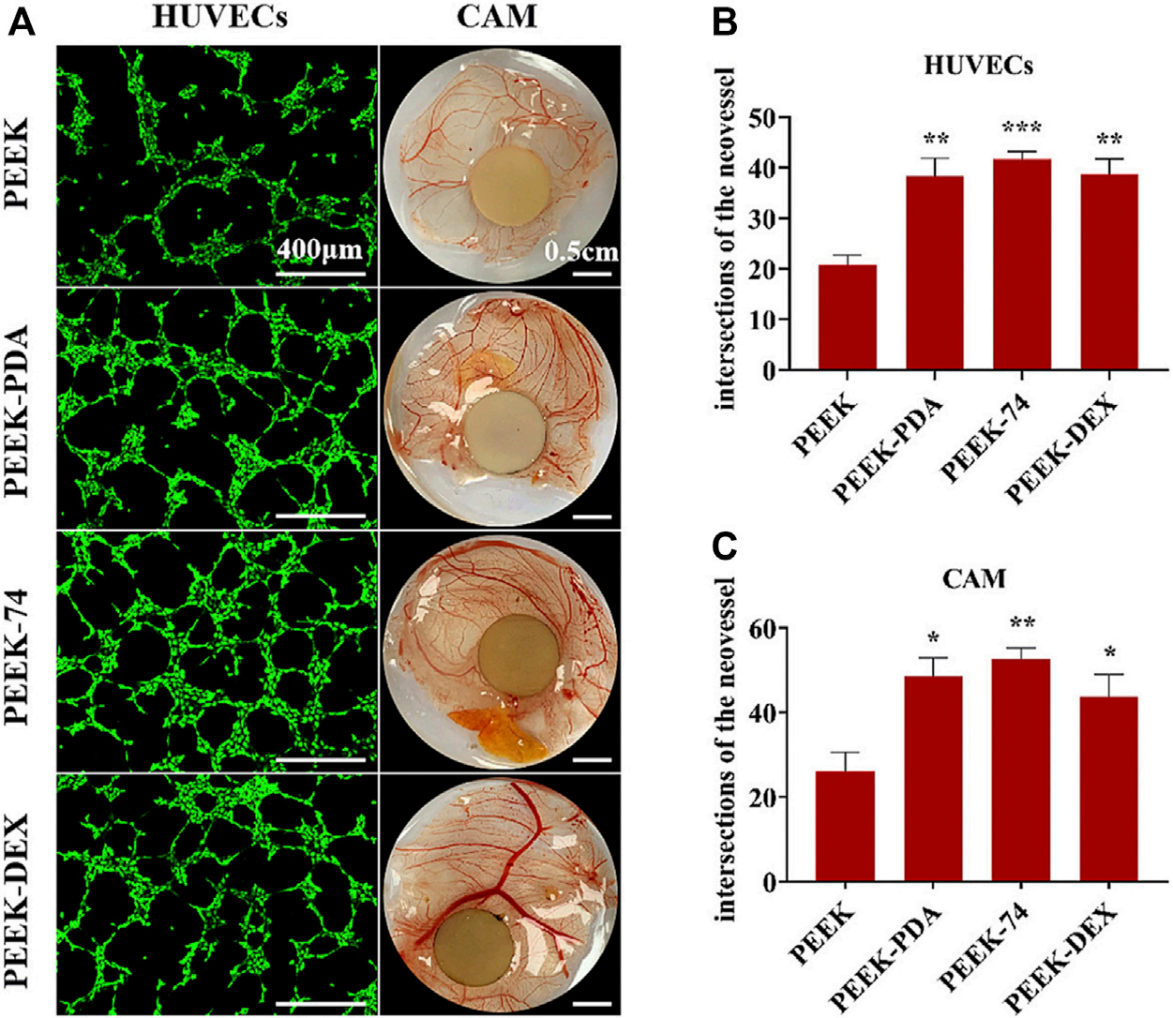
Blood vessel formation of different PEEK samples. **(A)** Pictures of HUVECs angiogenesis assay and vivo CAM assay; the number of intersections of the neovessel network: **(B)** HUVECs and **(C)** CAM; *p < 0.05, **p < 0.01, and ***p < 0.001 (Xiao et al., 2021). Copyright 2021, American Chemical Society.

VEGF is one of the most important regulators of angiogenesis, which is also significant for bone regeneration. Other bioactive components, such as bFGF, metallic ions, citrate and so on, also exhibit angiogenesis property. When applied in PDA-based multilayered coatings, the osteogenic activity will be synergistically enhanced and the improved new bone formation can be observed. Xiao et al. (Xiao et al., 2021) developed a dual-metal-organic framework (Zn-Mg-MOF74) coating on PEEK substrate *via* PDA intermediate layer, and loaded dexamethasone on it. The as-prepared multilayered coating exhibited strong antibacterial, angiogenic and osteogenic capacities. It improved angiogenic ability of human umbilical vein endothelial cells (HUVECs), as well as osteogenic differentiation of rat BMSCs. The *in vivo* chicken chorioallantoic membrane (CAM) model further demonstrated the outstanding angiogenesis of this multilayered coating (Figure 9). And rat femoral drilling model also verified the enhanced new bone formation with stronger bone-implant contact. In recent years, PEEK has attracted increasing attention as a promising, alternative, polymeric material for bone defect repair with lower Young’s modulus similar to human cortical bone, biocompatibility, chemical stability and radiation transparency. However, the bioinert properties and inferior osseointegration of PEEK surface severely hinder its universality of clinical application. The modification of PDA-based multilayered coating can provide a promising strategy for better bone formation on PEEK surface.

#### Antibiosis Property

After implantation, bacterial-related infection is a threating factor for bone regeneration, which includes bacterial adhesion and biofilm formation. Due to the antibiotic resistance and immune evasion, the bacterial-related infection is an important cause of treatment failure (Xie et al., 2019). Direct infection of bone cells can induce the secretion of osteoclastogenic cytokines and contribute to pathological bone loss. Furthermore, osteocytes are possible to serve as a long-term and immune-privileged reservoir for bacterial colonization, which come from differentiation or maturation of intracellularly infected osteoblasts. The inhibition of bacterial-derived infection can not only promote osseointegration after implantation, but also effectively prevent long-term infection (Masters et al., 2022). As a result, a desired scaffold for new bone formation should exhibit both antibacterial and osteogenic property.

**FIGURE 10:**
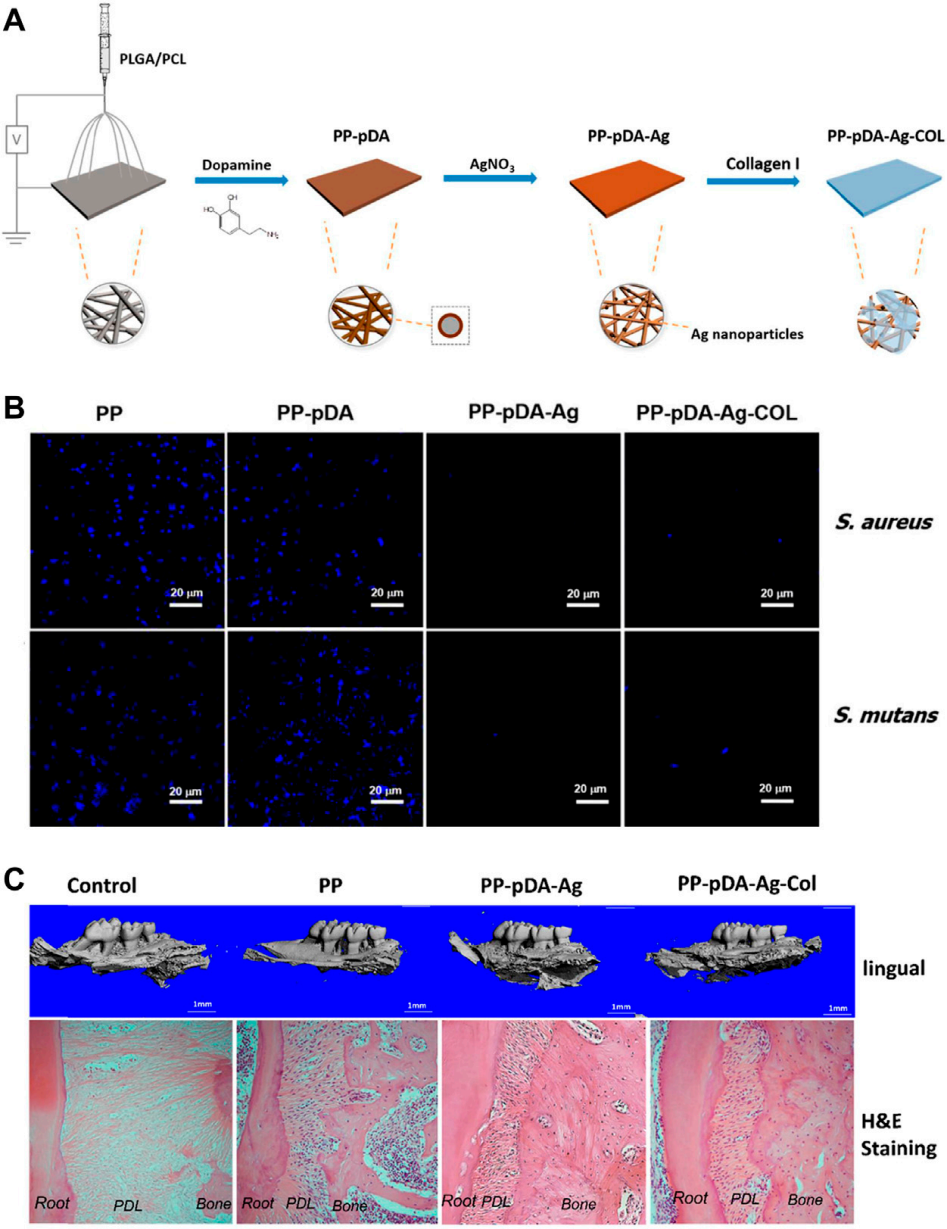
PDA-based PP-pDA-Ag-COL scaffold for antibacterial and osteogenic activity. **(A)** Schematic illustration of the preparation procedure. **(B)** DAPI immunofluorescent staining of bacterial attachment. **(C)** 3D reconstruction and H&E staining of the regenerated periodontal tissues in periodontitis mouse model 6 weeks after implantation (Qian et al., 2019). Copyright 2019, American Chemical Society.

The common components used for antibiotic application are metallic ions and antibiotics, such as Ag^+^, Cu^2+^, vancomycin gentamicin chlorhexidine and so on. Other antibacterial components, like proteins and peptides, attract relatively less attention, which are also effective for antibacterial applications. Considering the cytotoxicity of rapid release of antibacterial metallic ions, the PDA-based multilayered coatings allow introduction of favorable carriers to achieve long-term drug release. Qian et al., 2019) combined COL I and Ag nanoparticles to prepare a multifunctional layer with drug release capability on PDA-modified PLGA/PCL scaffolds. The PLGA/PCL scaffold, as synthetic polymers, can be fabricated with customized shape, pore size, porosity, degradation rate and mechanical properties to match the individual demands. The limitations mainly lie in their hydrophobic surface properties, which will inhibit cell attachment. Therefore, secondary surface modification is necessary to allow further clinical application. In this research, the multilayered coating exhibited satisfying antibacterial property, upregulated osteogenesis-related gene expression of MC3T3 cells, and enhanced alveolar bone regeneration in periodontal defects of mouse periodontal disease models (Figure 10).

#### Antitumor Property

After resection of bone tumors, the resultant bone defects will largely influence patients’ life quality. It is beneficial to carry out bone reconstructions *via* tissue engineering, but the tumor cells are hard to remove completely and the local relapse can occur due to residual tumor cells, which will have negative effects on bone regeneration and even lead to failure due to the poor bone reconstruction for tumor-related bone defects (Shao et al., 2021). Therefore, in order to repair bone defects after tumor section, the scaffolds should exhibit antitumor and osteogenic property.

**FIGURE 11:**
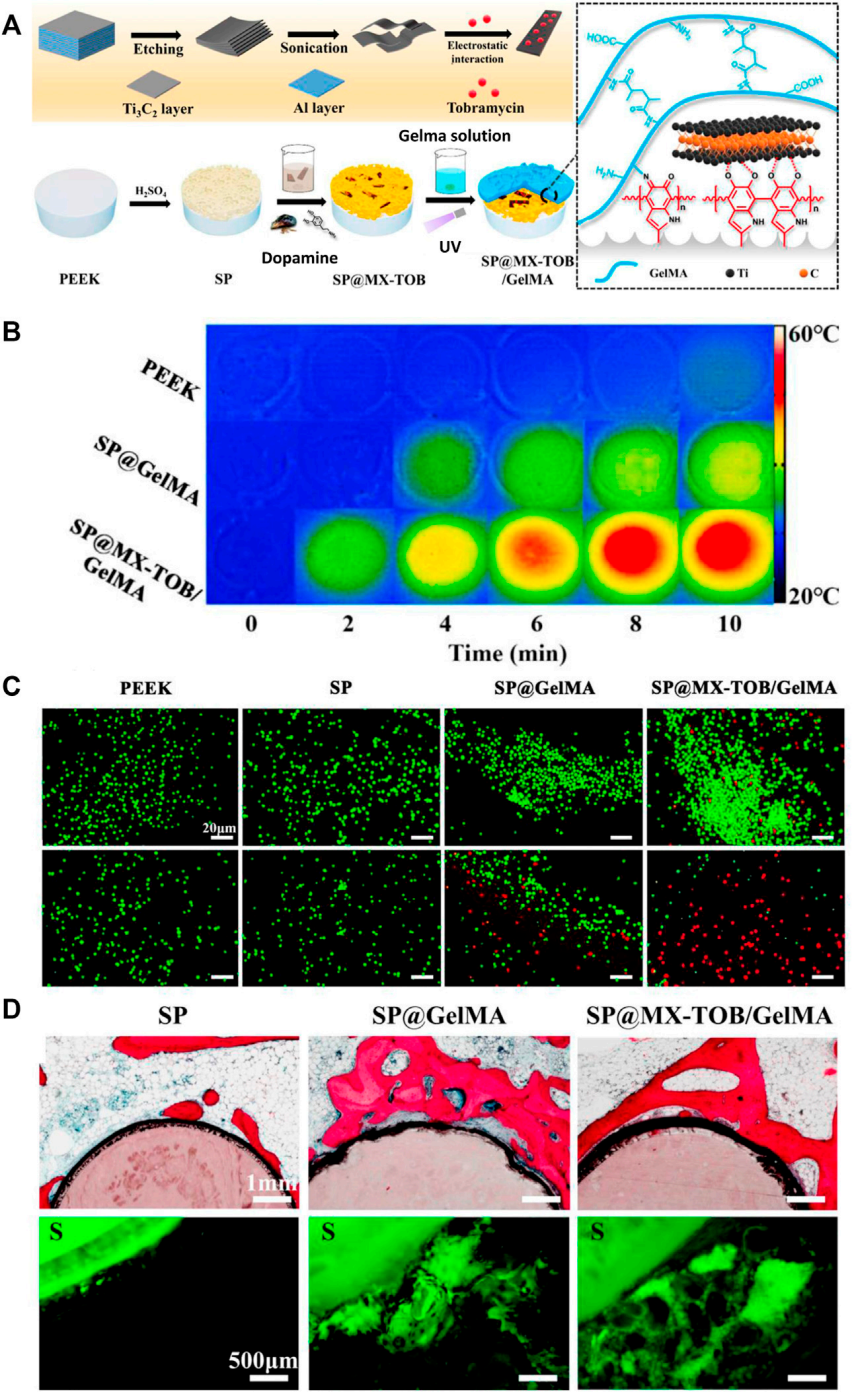
The PDA-based multilayered coating with multiple functions for tumor suppression and osteogenesis. **(A)** Schematic illustration of the coating fabrication. **(B)** Photothermal heating curves of different samples. **(C)** Live/Dead fluorescent staining of tumor cells. **(D)** *In vivo* evaluation of osteogenic activity. **p < 0.05 and **p < 0.01 (Yin et al., 2020). Copyright 2020, American chemical society.

Due to the similar structure to melanin, PDA exhibits photothermal property, whose conversion efficiency under near-infrared (NIR) is about 40%. Notably, NIR-induced photothermal therapy is considered a promising strategy for tumor treatment without damaging surrounding tissues (Ma et al., 2016; Shao et al., 2021). Meanwhile, due to the functional groups of PDA, antitumor drugs can be loaded in multilayered coatings, which can be released in a sustained manner. As a result, the PDA-based multilayered coatings with antitumor drugs can be applied to repair bone defects caused by tumors, which are able to avoid tumor relapse and enhance bone formation. Yin et al. (Yin et al., 2020) develop a PDA-mediated multilayered coating on sulfonated PEEK substrate, including MXene nanosheets and gelatin methacrylate (GelMA) hydrogels, in order to facilitate tumor cell death and enhance osteogenesis for patients after osteosarcoma resections (Figure 11). Due to the synergistic photothermal activity of PDA and MXene, this coating could effectively kill osteosarcoma cells *via* 808 nm NIR irradiation. Furthermore, through *in vitro* and *in vivo* experiments, it was demonstrated that the multilayered coating had superior cytocompatibility and osteogenesis activity, and exhibited antibacterial property when loading tobramycin.

## Conclusions and Outlooks

Thanks to its adhesive property, the PDA components can be immobilized to surfaces of all biomaterials, and makes it feasible to carry out secondary treatments to achieve PDA-based multilayered coatings. Besides osteogenic activity, these coatings often exhibit multifunctional property, like immunomodulation, angiogenesis, antibiosis, antitumor and so on. Therefore, PDA-based multilayered coatings seem to be a general strategy of surface modification for bone tissue repair, where the coating components can be adapted flexibly according to practical demands in different application scenarios.

However, the translations of these techniques into clinics still have a long way to go and there are several issues that should be addressed.

1) Based on the PDA intermediate layer, plenty of techniques have been developed to form multilayered coatings, among which there are considerable researches with similar techniques. Therefore, it will be helpful to make a systematic analysis of these researches and summarize a general strategy for certain techniques. In another word, before clinical application, it is necessary to normalize different techniques for better evaluation.
2) Compared with *in vitro* experiments, fewer *in vivo* experiments have been carried out, and few clinic trials of PDA-based multilayered coatings can be found. Even though it is important to carry out more basic researches to enhance bone formation for patients, it is of great significance to make efforts for clinical translation and more clinic trials should be scheduled.
3) As for basic researches, more efforts should be made to achieve a deeper understanding of the whole process and mechanism of bone formation on the PDA-based multilayered coatings. Besides osteogenic differentiation and osseointegration, more biological behaviors can be studied, including responses of different immune cells, long-term stability, *in vivo* pharmacokinetics and so on.

In conclusion, mussel-inspired PDA-based multilayered coatings seem a universal strategy for modification of biomaterials. The outstanding adhesive property of the PDA intermediate layer allow immobilization of addictive multifunctional components. The as-prepared PDA-based multilayered coatings often exhibit multifunctional property, which can meet different clinical demands in various application scenarios and synergistically enhance new bone formation. Nevertheless, it still needs more effort to translate these modification strategies into clinics.
